# A Model to Define Reference Ultrasound Parameters for Early Assessment of Nephron Endowment in Extremely Low Birth Weight Preterm Infants

**DOI:** 10.3390/children13050590

**Published:** 2026-04-24

**Authors:** Gabriele Villani, Raffaella Lamparelli, Carmelo Geraci, Gianfranco Maffei

**Affiliations:** Neonatal Intensive Care Unit, Azienda Ospedaliero Universitaria Ospedali Riuniti, 71122 Foggia, Italy; rlamparelli@ospedaliriunitifoggia.it (R.L.); cgeraci@ospedaliriunitifoggia.it (C.G.); gmaffei@ospedaliriunitifoggia.it (G.M.)

**Keywords:** preterm infants, extremely-low-birthweight, nephron endowment, reference ultrasound parameters

## Abstract

**Highlights:**

**What are the main findings?**

- This study proposes reference ultrasound parameters for the early assessment of nephron endowment in extremely low birth weight preterm infants. Based on the analysis of a cohort of 52 newborns, a model was developed using combined kidney volume indexed to body weight, as measured by ultrasound.
- This marker may reflect interindividual variability in renal development and pro-vide an early estimate of nephron endowment in this high-risk population.

**What are the implications of the main findings?**

- The proposed approach may enable the early identification of extremely low birth weight infants at higher risk of future renal impairment.
- The use of kidney volume indexed to body weight could support early risk strati-fication and guide personalized follow-up strategies, with the aim of improving long-term renal monitoring and preventing chronic kidney disease.

**Abstract:**

**Background**: Preterm birth, the leading cause of neonatal mortality, is associated with reduced nephron endowment and an increased risk of kidney disease in later life. In preterm infants, the interruption of nephrogenesis leads to a lower nephron number and structural abnormalities. Prenatal factors such as intrauterine growth restriction, and postnatal factors including nephrotoxic medications, patent ductus arteriosus, perinatal asphyxia, and infections contribute to this deficit. Ultrasound is a key tool for assessing renal volume at birth and can, when indexed to body weight, be used to estimate nephron endowment, which is known to vary widely among individuals. **Methods**: This study analyzed 52 preterm infants with birth weight < 1000 g, assessing combined renal volume (sum of right and left kidney volumes) indexed to body weight. **Results**: The mean combined kidney volume-to-body weight ratio was 12.12 (SD = 2.03). Values below the 10th percentile (9.46) or more than one standard deviation below the mean (10.11) may indicate nephron deficiency at birth. **Conclusions**: Standardized ultrasound-based parameters enable the early identification of neonates at risk for nephron deficit, supporting targeted preventive strategies. Long-term follow-up is essential to detect early renal functional impairment and reduce the risk of chronic kidney disease.

## 1. Introduction

Preterm birth, defined as delivery before 37 weeks of gestational age, remains the leading cause of neonatal mortality. In recent decades, advances in perinatal care have significantly improved the survival of preterm infants; however, many still experience both short- and long-term morbidities, inversely correlated with gestational age and birth weight [1,2]. Among the organs most affected are the kidneys, whose development begins around the fifth week of gestation and continues until approximately the 32nd to 36th week, resulting in a total nephron count ranging from 200,000 to over 2 million. Nephrogenesis may continue for an additional 4 to 6 weeks after birth [3,4]. In premature infants, early birth disrupts normal nephrogenesis, leading to a reduced nephron number and the development of structurally and functionally abnormal nephrons [5]. Prenatal insults, such as intrauterine growth restriction (IUGR) [6], and postnatal factors occurring during admission to the Neonatal Intensive Care Unit (NICU), further contribute to the reduction in nephron endowment. These include the use of nephrotoxic drugs, perinatal asphyxia, hypotensive episodes, severe infections, hypoxia, acute kidney injury (AKI), and hemodynamic instability due to a patent ductus arteriosus (PDA) or its treatment [7,8]. Consequently, preterm infants, who are genetically predisposed to a lower nephron number, are more vulnerable to developing chronic kidney disease compared to those with a greater nephron endowment. Compensatory glomerular hypertrophy and hyperfiltration have been associated with an increased risk of hypertension, cardiovascular disease, and renal failure, particularly during adolescence and adulthood [9,10]. Early identification of preterm infants with reduced nephron endowment is therefore essential to enable appropriate monitoring of short-, medium-, and long-term outcomes. Ultrasound is the imaging modality of choice for assessing renal size as it is noninvasive, free of ionizing radiation, cost-effective, and easily performed at the bedside [11,12]. In a recent study, we observed that combined renal volume (CRV)—defined as the sum of the right and left kidney volumes measured by ultrasound—correlates strongly with body weight from one week after birth to 24 months of postmenstrual age (PMA) in extremely low birth weight (ELBW) preterm infants. This correlation is stronger with body weight than with neonatal length, likely due to the greater accuracy of electronic scales integrated into modern incubators. These findings suggest that renal volume indexed to body weight may serve as a reliable marker of nephron endowment at birth [13]. With regard to normalization to body surface area (BSA), although this approach has been proposed in the literature, it presents significant practical limitations in the neonatal setting. In particular, accurate measurement of body length—required for BSA calculation—is often challenging and unreliable in very low gestational age infants due to posture, fragility, and clinical instability. For this reason, several authors have suggested that body weight alone represents a more feasible and appropriate parameter for clinical application [14].

Building on this study, the present work suggests a model to define reference ultrasound parameters for the early assessment of nephron endowment in extremely low birth weight preterm infants, with the aim of informing a more targeted and individualized follow-up strategy.

## 2. Materials and Methods

### 2.1. Participants and Study Design

The initial cohort of 29 ELBW preterm infants born between March 2018 and March 2021 at the Ospedali Riuniti University Hospital (Foggia, Italy) was expanded by including 23 additional infants admitted to the same Neonatal Intensive Care Unit with similar characteristics. Caffeine was used for the prevention and treatment of apnea of prematurity, according to current neonatal guidelines. The standard regimen included a loading dose of 20 mg/kg (caffeine citrate), followed by a maintenance dose of 5–10 mg/kg/day [15]. Aminoglycosides were administered for suspected/confirmed sepsis, with dosing adjusted for gestational and postnatal age and guided by therapeutic drug monitoring [16]. Hemodynamically significant patent ductus arteriosus (hsPDA) was defined based on combined echocardiographic and clinical criteria. Echocardiographic parameters included a ductal diameter ≥ 1.5 mm, a left atrium-to-aortic root ratio (LA/Ao) ≥ 1.5, evidence of increased pulmonary blood flow or systemic hypoperfusion (e.g., reversed diastolic flow in the descending aorta or mesenteric arteries), and a predominantly left-to-right shunt with a low-velocity pulsatile Doppler pattern. These findings were integrated with clinical signs such as the need for respiratory support, hypotension, widened pulse pressure, and pulmonary overcirculation [17]. When treatment was indicated, intravenous ibuprofen was administered at an initial dose of 10 mg/kg, followed by 5 mg/kg at 24 and 48 h [18]. Hemodynamic support with fluids and, when indicated, inotropes maintained systemic blood pressure within acceptable limits. Serum creatinine (SCr) was measured in all neonates no earlier than the third day of life to minimize maternal value interference. Measurements were performed using the Jaffé colorimetric assay in the centralized and standardized clinical laboratory of our institution, using the modified neonatal KDIGO (nKDIGO) [19,20] criteria as reference standards. In neonates, these criteria do not define fixed reference values for SCr; instead, acute kidney injury is classified based on relative increases from baseline creatinine levels. Urine output was quantified by diaper weight or by using a neonatal urine collection bag. Measurements were recorded daily and expressed in milliliters per kilogram per hour (mL/kg/h).

### 2.2. Measurements

The right and left renal volumes, measured ultrasonographically in cubic centimeters (cm^3^) approximately one week after birth (±1 day) to allow for clinical stabilization, were summed to calculate the combined renal volume of each infant. Body weight was recorded at the time of the ultrasonographic assessment. Ultrasound examinations were performed using a scanner equipped with a convex abdominal probe (5–8 MHz) and ultrasound gel pre-warmed to body temperature. All examinations were conducted by an experienced examiner to minimize interobserver variability. With the neonate in the prone position, renal length was measured in the sagittal plane as the maximum longitudinal cranio-caudal distance. The antero-posterior and transverse diameters of the kidney were measured in an axial plane, where the organ appeared symmetrically round, with all dimensions recorded perpendicularly. Ultrasound images were appropriately magnified to ensure accurate measurements. Renal volume was calculated using the ellipsoid formula: volume = length × width × depth × 0.523 [21,22]. Although more advanced techniques, such as image segmentation, may provide potentially more accurate measurements, we deliberately adopted the ellipsoid method as it remains the most widely used approach in both term and preterm neonates in clinical practice. Its broad applicability is primarily related to its simplicity, good reproducibility, and feasibility in bedside settings. This approach is consistent with previous studies in neonatal populations [23,24,25,26]. In this context, the use of the ellipsoid formula was considered an appropriate methodological choice, representing a practical balance between measurement accuracy and clinical feasibility. The ultrasound examination was performed at the patient’s bedside as part of a point-of-care ultrasound (POCUS) program in the NICU. This approach requires specific skills but allows for rapid and practical execution [27]. Two-dimensional ultrasonography (2D-US) with the ellipsoid formula was selected instead of three-dimensional ultrasonography (3D-US) because it is widely used in neonatal and pediatric studies and offers greater feasibility in clinical practice. It provides a reasonable approximation of renal volume despite its known limitations compared with 3D-US, which offers a more accurate representation of renal morphology. From a research perspective, the use of 3D-US may serve as a basis for future studies aimed at validating the proposed cutoff values and standardizing reference curves. Body weight was measured using an electronic scale integrated into the incubator. In ELBW infants, ensuring adequate hydration is a fundamental aspect of good clinical practice. To this end, a combined strategy of enteral and, when necessary, parenteral nutrition is used, together with continuous monitoring of hydration status [28,29,30]. This approach enables body weight to be used as a reliable parameter for indexing renal volume.

### 2.3. Protocol and Data Analysis

The original study protocol was approved by the Ethics Committee of the Azienda Ospedaliero-Universitaria Ospedali Riuniti di Foggia, Italy (Ref. 106, Opinion No. 54/CE/2018; approved on 6 March 2018). The study was conducted in accordance with the Declaration of Helsinki and its subsequent amendments. Informed consent, including details of the study and authorization to participate and for data publication, was obtained from the participants’ parents. Data were analyzed using commercial software (SPSS, version 11.0, 2002; SPSS Inc., Chicago, IL, USA). Combined renal volume was indexed to body weight (in kilograms) for each of the 52 ELBW infants. The normality of the data distribution was assessed using the Shapiro–Wilk test. A *p*-value < 0.05 was considered statistically significant. Z-scores were calculated for each CRV value indexed to body weight. Values were grouped into the 10th, 25th, 50th, 75th, and 90th percentiles, and categorized according to Z-score ranges: within ±1 SD, ±2 SD, and >2 SD.

The objective of this study is descriptive rather than aimed at developing reference curves (centiles for predictive or clinical purposes), which would require larger, preferably multicenter, cohorts. Given the symmetrical distribution of the data and the satisfactory sample size, the most commonly used centiles (10th, 25th, 50th, 75th, and 90th) were reported, whereas the extreme centiles (e.g., 3rd or 97th) were not included, as their estimation would require several hundred observations to ensure statistical reliability. The standard deviation calculated from 52 observations can be considered appropriate for a clinical research setting, particularly within the context of a single-center, time-limited observational study conducted in a highly selected population, such as preterm infants with a birth weight below 1000 g. Other data are presented as minimum (Min), maximum (Max), median (Mdn), interquartile range (IQR), mean (M), and standard deviation (SD).

## 3. Results

A total of 52 extremely low birth weight infants (birth weight ≤ 1000 g) were included in the analysis, comprising both males and females and including infants born either inborn or outborn. Infants with malformative nephrouropathies detected on fetal or neonatal ultrasound, major congenital malformations, or genetic and chromosomal syndromes were excluded. According to the Italian Neonatal Study (INeS) growth charts [31], 10 infants were classified as small for gestational age (SGA), 1 as large for gestational age (LGA), and the remaining 41 as appropriate for gestational age (AGA). Seventeen infants were male and thirty-five were female. Gestational age at birth ranged from 23 + 3 to 32 + 0 weeks, with a median of 26 + 4 weeks (interquartile range-IQR, 4). Birth weight ranged from 300 to 1000 g, with a median of 810 g (IQR, 245). Forty-five infants received antenatal betamethasone prophylaxis for the prevention of hyaline membrane disease. Fifteen had birth asphyxia documented by blood gas analysis performed within 1 h of birth, based on the following criteria: Apgar score < 5 at 5 min and/or pH < 7 and/or base excess (BE) > −12), and forty-three received at least one dose of endotracheal surfactant. During their NICU stay, all neonates received noninvasive ventilation, and 37 also required invasive mechanical ventilation. Four neonates did not require oxygen therapy. All infants were treated with xanthines and aminoglycosides. Fourteen experienced hemodynamic compromise due to patent ductus arteriosus while 15 developed serious infections. SCr levels ranged from 0.1 to 2.58 mg/dL, with a median of 0.63 (IQR, 0.67). One neonate had a maximum SCr of 1.53 mg/dL on a single occasion, another reached 1.84 mg/dL once, and a third reached 2.58 mg/dL, all without subsequent elevations. During hospitalization, urine output ranged from 2.21 to 6.03 mL/kg/h, with a median of 3.77 mL/kg/h (IQR, 1.30).

Among the 52 ELBW newborns, combined renal volumes indexed to body weight ranged from a minimum of 7.7 to a maximum of 18.07. The mean was 12.12 (SD = 2.03), while the median was 12.06 (IQR = 2.52).

Table 1 shows the data collected for each newborn one week after birth, including gestational age (expressed in weeks and days), combined renal volume, body weight (kilograms), and the ratio of combined renal volume to body weight with the corresponding Z-score.

The Shapiro–Wilk test did not indicate a significant departure from normality, W(52) = 0.98, *p* = 0.377. This suggests that the data did not significantly deviate from a normal distribution. The test statistic W was 0.9761, which falls within the 95% confidence interval [0.9555, 1]. The *p*-value of 0.377 provides insufficient evidence to reject the null hypothesis. The observed effect size (KS–D) was small (0.096). Despite the presence of an outlier (18.07), the test does not provide sufficient evidence to reject the assumption of normality. The Shapiro–Wilk test, designed for small to moderate sample sizes, is appropriate for a sample of *n* = 52, providing sufficient power to detect potential deviations from normality. Therefore, the 52 available observations represent an adequate sample size for the reliable application of this test.

**Figure 1:**
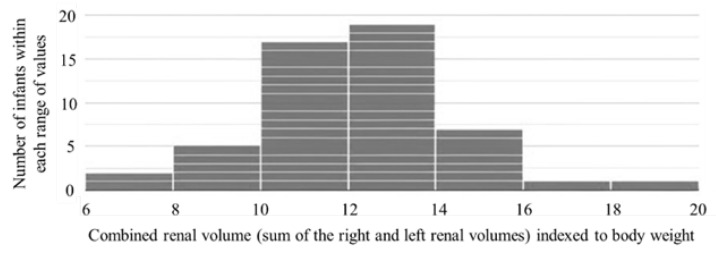
Distribution of combined renal volumes indexed to body weight (cm^3^/kg) one week after birth in 52 ELBW preterm infants.

The histogram illustrates the distribution of combined renal volumes indexed to body weight (cm^3^/kg) one week after birth in 52 ELBW preterm infants. The *x*-axis represents the combined renal volume—defined as the sum of the right and left renal volumes, indexed to body weight—while the *y*-axis indicates the number of infants within each range of values (Figure 1).

**Figure 2:**
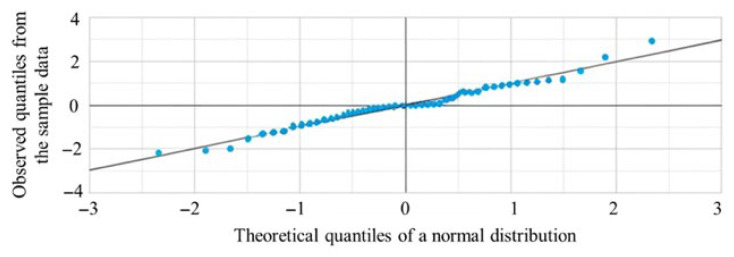
Distribution of combined renal volumes indexed to body weight (cm^3^/kg) one week after birth in 52 ELBW preterm infants. Most data points aligned with the reference line, suggesting an approximately normal distribution, with minor deviations observed in the tails.

The Q–Q plot displays the distribution of combined renal volumes indexed to body weight one week after birth in 52 ELBW preterm infants. The *x*-axis represents the theoretical quantiles of a normal distribution, and the *y*-axis represents the observed quantiles from the sample. Most data points fell along the reference line, indicating an approximately normal distribution, with minor deviations observed in the tails (Figure 2). The Q–Q plot does not require a strict minimum sample size threshold: with 52 data points, it is possible to clearly identify non-normal patterns such as skewness, heavy tails, or outliers. The plot was sufficiently dense to provide meaningful visual information; also, in this case, 52 observations were more than adequate for a reliable assessment.

The combined renal volume indexed to body weight was 9.46 at the 10th percentile, 10.87 at the 25th percentile, 12.06 at the 50th percentile, 13.39 at the 75th percentile, and 14.39 at the 90th percentile (Table 2).

Values within one standard deviation below the mean ranged from 10.11 to 12.12. Those between one and two standard deviations below the mean ranged from 8.09 to 10.11, while values more than two standard deviations below the mean were less than 8.09. Similarly, the categories above the mean included values within one standard deviation ranging from 12.12 to 14.14; those between one and two standard deviations above the mean range from 14.14 to 16.16; and values more than two standard deviations above the mean were greater than 16.16 (Table 3).

## 4. Discussion

Chronic kidney disease often progresses silently, with clinical signs typically emerging only in advanced stages, thus complicating early detection. Given that prematurity is a risk factor per se, preterm infants with reduced nephron numbers are particularly vulnerable and require more rigorous clinical monitoring. However, individual variability in nephron endowment and the absence of standardized methods for its measurement pose significant challenges for early identification and risk stratification. Exposure to nephrotoxic drugs, the presence of PDA, severe infections, and the need for mechanical ventilation represent common clinical conditions in extremely low birth weight preterm neonates admitted to the NICU. These factors may influence both nephron endowment and the long-term trajectory of renal function, as previously highlighted in our earlier study [13]. However, these variables were not included in the statistical adjustment of the present analysis, as this was beyond the primary scope of the study. The main objective was to propose an ultrasound-based model for defining reference parameters that may enable the early estimation of nephron endowment in this high-risk population. Our intent was to propose a model for defining ultrasound-based reference parameters useful for the early estimation of nephron endowment in extremely low birth weight preterm neonates, so that single-center or multicenter studies conducted on larger populations may provide reference values generalizable to this entire patient category, rather than to exhaustively analyze the clinical determinants of renal volume variability.

Numerous studies have documented that preterm birth may impair nephrogenesis, thereby increasing the risk of hypertension and chronic kidney disease in adulthood [32,33,34]. Additional evidence indicates that preterm infants have significantly smaller renal parenchyma and total kidney volume compared to their full-term counterparts at the same corrected gestational age. This reflects a reduced nephron endowment, which is partially compensated by hyperfiltration—an adaptive mechanism that may be associated with glomerular injury and a higher risk of long-term renal dysfunction [11,35].

According to several authors, cystatin C is considered superior to GFR in assessing both renal function and tubular rearrangement, particularly in relation to reduced glomerular number [36,37]. Moreover, Aisa et al. (2016) emphasized that studies investigating renal physiology in preterm neonates and/or those with intrauterine growth restriction rarely included correlation analyses between biochemical parameters and nephron number, although such analyses, according to the authors, could provide additional insights and facilitate the early identification of individuals at higher risk of renal insufficiency [38].

Several recent studies have shown a weak correlation between indirect measures of nephron mass and biomarkers such as cystatin C, creatinine, and albuminuria, indicating that these markers are not sufficiently sensitive to detect early reductions in renal function associated with decreased nephron endowment [39,40,41]. For this reason, our study aims to provide only ultrasonographic reference values for estimating nephron endowment at birth.

Recent studies suggest that for an accurate assessment of kidney growth and health, measuring kidney volume is preferable to relying exclusively on kidney length. Although length is easily accessible—also through freely available applications for calculating Z-scores—it may lead to overestimations unrelated to age and may not accurately reflect renal development. This limitation was demonstrated by Torres-Canchala et al., who reported a weak correlation between length Z-scores and kidney volume (r = 0.32), along with a significant bias in the Bland–Altman analysis. Moreover, Scholbach et al. proposed using kidney volume indexed to body surface area (BSA) as a reliable and universally applicable parameter, normally distributed across all ages, to monitor renal function over time and to identify pathological deviations without the need for age-specific reference tables. However, the study by Scholbach et al. did not include preterm infants, for whom BSA—calculated based on length—is less accurate than body weight [42,43].

In our previous study, we introduced an innovative approach to estimating nephron endowment at birth in extremely low birth weight preterm infants by indexing renal volume to body weight.

In that study, we observed substantial inter-individual variability in both kidney volume and body weight among preterm neonates, even within the same gestational age group. This variability further supports the notion that renal size alone is a poor surrogate for nephron endowment. Notably, although CRV and body weight were strongly correlated at the population level, this relationship was also consistently maintained within each individual infant over time, from the first week of life through 24 months of PMA. This longitudinal stability suggests that kidney growth closely parallels somatic growth, rather than reflecting differences in nephron number.

The seventh day of life was selected to allow for greater clinical stabilization and to ensure safe conditions in extremely preterm neonates, who are particularly fragile in the immediate postnatal period. We agree that body weight on the seventh day may be influenced by physiological postnatal fluid loss. This aspect was carefully considered from the early stages of the study. Specifically, we observed that the correlation between combined kidney volume and body weight was consistently high in each infant from the first assessment performed within the first week of life and remained stable in subsequent measurements up to 24 months of postmenstrual age [13].

This finding suggests that early postnatal fluid loss does not substantially compromise the reliability of the combined kidney volume-to-body weight ratio. It is plausible that this robustness is also attributable to appropriate fluid management and standardized neonatal intensive care practices.

The findings suggest that absolute renal volume, which varies considerably even among infants of the same gestational age, does not accurately reflect nephron endowment at birth. In contrast, the renal volume-to-body weight ratio appears to provide a more reliable estimate of nephron mass [13]. We did not perform a separate analysis for SGA, AGA, and LGA neonates, as the strength of the correlation between combined kidney volume and body weight was comparable and consistently high across all three categories. This finding suggests that the observed association is not significantly influenced by growth status at birth, including intrauterine growth restriction.

The present observational study, while confirming that ultrasound is the most appropriate method for measuring renal volume at birth in ELBW preterm infants, takes a further step by providing useful reference parameters for assessing nephron endowment in this vulnerable population. This may allow for the early identification of infants with reduced nephron endowment and support the planning of more targeted follow-up strategies.

A limitation of this study is the small sample size. Extremely low birth weight preterm infants represent a numerically limited population, making data collection challenging in single-center studies. This research may serve as a model and a stimulus for interinstitutional collaboration, encouraging the development of multicenter studies to establish universal benchmarks.

In addition, collecting data on renal development during the first years of life would allow for the correlation of neonatal ultrasound parameters with long-term renal outcomes. Renal volume indexed to body weight at birth could also serve as a reference for other groups of preterm and full-term infants. To enhance clinical relevance, future studies should aim to include a larger sample size and incorporate long-term follow-up to assess the actual impact on renal health in adulthood.

## 5. Limitations

This study has several limitations that should be carefully considered. First, studies assessing renal volume in extremely preterm infants are scarce, and normative reference values for this population are currently lacking. Although some authors have correlated ultrasound-derived renal measurements with inulin clearance or two-point plasma clearance, such data are not available for extremely low birth weight (ELBW) infants.

The first days of life in extremely preterm infants are characterized by hemodynamic instability and rapid fluctuations in fluid balance, both of which may influence ultrasound measurements of renal volume. Despite recognizing the importance of these factors, we deliberately chose not to perform serial ultrasound assessments on days 1 and 7. Instead, ultrasound evaluation was performed exclusively on day 7 to minimize the risks associated with handling extremely fragile infants at the borderline of viability (gestational age < 25 weeks and birth weight < 600 g), belonging to the ELBW category. Therefore, the absence of an early (day 1) ultrasound assessment represents a limitation driven by safety considerations, particularly for the smallest and most vulnerable patients. Future studies, possibly involving less premature populations, may consider performing serial ultrasound assessments from birth through the early postnatal period.

An additional limitation is that only a single measurement was obtained on day 7 of life, without prior serial assessments or objective markers of hydration status. Although hydration was carefully managed and monitored in clinical practice, as described in Section 2, the absence of serial measurements prevents complete exclusion of the potential influence of early fluid shifts on renal ultrasound findings.

Furthermore, the criterion used to estimate nephron endowment at birth in this cohort was derived from a previously published study, cited herein, which demonstrated a strong correlation between combined renal volume and body weight. Accordingly, renal volume indexed to body weight, rather than absolute renal volume, may represent a more appropriate surrogate of nephron endowment. In the study entitled *Kidney* *volume-to-birth weight ratio as an estimate of nephron endowment in extremely low birth weight preterm infants* (Scientific Reports, 2024), a total of 224 renal ultrasound scans were performed at predefined time points: from the seventh day of life to 38–40 weeks postmenstrual age, as well as during follow-up at 6, 12, 18, and 24 months of corrected age [13]. Although that study included 29 infants who are also part of the present cohort (*n* = 52), its longitudinal design partially mitigates the limitation of relying on a single early measurement, allowing day-7 data to be interpreted within a developmental trajectory rather than as an isolated observation.

Finally, another limitation is the lack of analyses adjusted for relevant clinical conditions, such as hemodynamically significant patent ductus arteriosus (hsPDA) and sepsis. This limitation primarily concerns hsPDA. Future studies, also in light of our findings, are warranted to better clarify the extent to which hsPDA may influence renal perfusion and renal volume in ELBW infants during the first week of life.

With regard to sepsis, most episodes in ELBW infants are late-onset and nosocomial, whereas early-onset sepsis represents a minority of cases, with variable incidence depending on the population studied and diagnostic criteria applied [44]. Therefore, it is reasonable to assume that within the first seven days of life—the time frame considered in the present study—the low incidence of sepsis is unlikely to have significantly influenced the mean values, standard deviations, or Z-scores of the sample.

## 6. Conclusions

In conclusion, this study, conducted in a cohort of 52 extremely low birth weight preterm infants, identified a subgroup of patients potentially at higher risk who may benefit from closer and individualized follow-up strategies. Specifically, infants presenting a combined renal volume-to-body weight ratio below 9.46 (10th percentile) or 10.11 (−1 SD), measured within the first week of postnatal life, appear to have a lower estimated nephron endowment. This finding supports the use of weight-indexed renal volume as a sensitive and clinically meaningful parameter for early risk stratification in this vulnerable population. The considerable interindividual variability observed in absolute renal volume measurements further underscores the importance of normalization to body weight in order to improve diagnostic accuracy. When performed by experienced operators using standardized protocols, renal ultrasound represents a non-invasive, reproducible, and clinically valuable tool for the early identification and longitudinal monitoring of ELBW infants. Importantly, the identification of this at-risk subgroup may enable the implementation of tailored surveillance programs aimed at the early detection and prevention of chronic kidney disease later in life. Nevertheless, larger multicenter studies are warranted to validate the proposed cut-off values and to confirm their applicability in broader clinical settings, ultimately supporting their integration into neonatal care pathways.

## Figures and Tables

**Table 1:** Gestational age, combined kidney volume, body weight, kidney volume-to-body weight ratio, and its Z-score in 52 ELBW preterm infants at one week of age.

GA	CRV	BW	Ratio	Z-Score	GA	CRV	BW	Ratio	Z-Score
24 + 3	4.61	0.443	10.41	−0.82	27 + 4	11.94	0.890	13.42	0.64
24 + 3	5.11	0.570	8.96	−1.53	27 + 5	4.97	0.630	7.89	−2.05
24 + 3	6.26	0.470	13.32	0.59	27 + 5	8.35	0.700	11.93	−0.08
24 + 5	4.06	0.337	12.05	−0.02	27 + 6	6.13	0.640	9.58	−1.23
25 + 2	9.38	0.670	14.00	0.93	28 + 0	8.97	0.703	12.76	0.32
25 + 4	6.59	0.610	10.80	−0.63	28 + 2	8.02	0.825	9.73	−1.16
25 + 4	4.08	0.530	7.70	−2.15	28 + 3	11.86	0.819	14.48	1.16
25 + 6	6.72	0.555	12.11	0.00	28 + 3	10.47	0.850	12.32	0.11
25 + 6	7.72	0.644	11.99	−0.05	28 + 3	12.85	0.930	13.82	0.84
26 + 1	10.91	0.660	16.53	2.16	29 + 0	8.46	0.735	11.51	−0.29
26 + 2	8.73	0.690	12.65	0.27	29 + 6	11.58	0.960	12.06	−0.02
26 + 2	7.10	0.580	12.24	0.07	29 + 6	10.59	1.000	10.59	−0.74
26 + 2	7.16	0.620	11.54	−0.27	30 + 0	9.17	0.751	12.21	0.05
26 + 2	8.92	0.630	14.16	1.00	30 + 2	9.82	0.835	11.76	−0.17
26 + 2	12.43	0.688	18.07	2.91	30 + 2	9.21	0.837	11.00	−0.54
26 + 4	9.27	0.641	14.46	1.15	30 + 2	8.89	0.867	10.26	−0.90
26 + 4	11.67	0.763	15.29	1.56	30 + 2	10.91	0.950	11.49	−0.30
26 + 5	10.08	0.732	13.77	0.81	30 + 2	7.75	0.640	12.11	0.00
26 + 6	8.76	0.658	13.32	0.60	30 + 3	8.69	0.923	9.41	−1.31
26 + 6	7.86	0.670	11.73	−0.18	30 + 4	10.59	0.885	11.97	−0.06
26 + 6	9.70	0.730	13.29	0.58	30 + 5	12.22	0.860	14.21	1.03
27 + 0	9.76	0.700	13.94	0.90	31 + 5	12.30	0.864	14.24	1.04
27 + 0	7.80	0.720	10.83	−0.62	31 + 6	9.84	0.815	12.07	0.01
27 + 1	6.59	0.512	12.87	0.38	32 + 4	10.73	1.032	10.40	−0.83
27 + 2	10.71	0.950	11.27	−0.40	32 + 6	11.40	0.935	12.19	0.04
27 + 3	5.13	0.440	11.66	−0.21	33 + 0	5.78	0.721	8.02	−1.99

Gestational age (GA) expressed in weeks and days (wk + d), combined renal volume (CRV) calculated as the sum of right and left kidney volumes (in cm^3^), body weight (BW) in kilograms, combined renal volume-to-body weight ratio (CRV/BW), and its Z-score, all measured at one week of postnatal age.

**Table 2:** Percentile distribution of combined renal volume indexed to body weight (CRV/BW) at one week of age in 52 ELBW preterm infants.

Percentiles	CRV/BW
Within 1st Standard Deviation	10.11 to 12.12
Within 2nd Standard Deviation	8.09 to 10.11
Beyond 2nd Standard Deviation	<8.09

Combined renal volume, defined as the sum of right and left renal volumes and indexed to body weight (CRV/BW), reported at the 10th, 25th, 50th, 75th, and 90th percentiles in 52 ELBW preterm infants evaluated at one week of age.

**Table 3:** Distribution of combined renal volume indexed to body weight (CRV/BW) by standard deviation categories in 52 ELBW preterm infants at one week of age.

CRV/BW	Values Below the Mean	Values Above the Mean
Within 1st Standard Deviation	10.11 to 12.12	12.12 to 14.14
Within 2nd Standard Deviation	8.09 to 10.11	14.14 to 16.16
Beyond 2nd Standard Deviation	<8.09	>16.16

Combined renal volume, defined as the sum of right and left renal volumes and indexed to body weight (CRV/BW), stratified by standard deviation ranges from the mean (within 1 SD, between 1 and 2 SD, and beyond 2 SD), in 52 ELBW preterm infants evaluated at one week of age.

## Data Availability

The original contributions presented in this study are included in the article. Further inquiries can be directed to the corresponding author.

## Abbreviations

The following abbreviations are used in this manuscript:

IUGR	Intra Uterine Growth Restriction
NICU	Neonatal Intensive Care Unit
AKI	Acute Kidney Injury
PDA	Patent Ductus Arteriosus
CRV	Combined Renal Volume
PMA	Post Menstrual Age
ELBW	Extremely Low Birth Weight
SGA	Small for Gestational Age
LGA	Large for Gestational Age
AGA	Appropriate for Gestational Age
KDIGO	Kidney Disease: Improving Global Outcomes
POCUS	Point-Of-Care Ultrasound
